# Spatial analysis of HIV infection and associated individual characteristics in Burundi: indications for effective prevention

**DOI:** 10.1186/s12889-016-2760-3

**Published:** 2016-02-04

**Authors:** Emmanuel Barankanira, Nicolas Molinari, Théodore Niyongabo, Christian Laurent

**Affiliations:** 1 Département des Sciences Naturelles, Ecole Normale Supérieure, Bujumbura, Burundi; 2 TransVIHMI, IRD UMI 233 / INSERM U 1175 / Université de Montpellier, Montpellier, France; 3 IMAG, UMR 519 / Centre Hospitalier Régional Universitaire de Montpellier / Université de Montpellier, Montpellier, France; 4 Centre Hospitalier Universitaire de Kamenge, Bujumbura, Burundi; 5 Institut de Recherche pour le Développement (UMI 233), 911 avenue Agropolis, BP 64501, Montpellier, 34394 cedex 5 France

**Keywords:** HIV, Prevalence, Heterogeneity, Spatial, Factors

## Abstract

**Background:**

Adequate resource allocation is critical in the battle against HIV/AIDS, especially in Africa. The determination of the location and nature of HIV services to implement must comply with the geographic, social and behavioral characteristics of patients. We therefore investigated the spatial heterogeneity of HIV prevalence in Burundi and then assessed the association of social and behavioral characteristics with HIV infection accounting for the spatial heterogeneity.

**Methods:**

We used data from the 2010 Demographic and Health Survey. We analyzed these data with a geostatistical approach (which takes into account spatial autocorrelation) by i) interpolating HIV data using the kernel density estimation, ii) identifying the spatial clusters with high and low HIV prevalence using the Kulldorff spatial scan statistics, and then iii) performing a multivariate spatial logistic regression.

**Results:**

Overall HIV prevalence was 1.4 %. The interpolated data showed the great spatial heterogeneity of HIV prevalence (from 0 to 10 %), independently of administrative boundaries. A cluster with high HIV prevalence was found in the capital city and adjacent areas (3.9 %; relative risk 3.7, *p* < 0.001) whereas a cluster with low prevalence straddled two southern provinces (0 %; *p* = 0.02). By multivariate spatial analysis, HIV infection was significantly associated with the female sex (posterior odds ratio [POR] 1.36, 95 % credible interval [CrI] 1.13-1.64), an older age (POR 1.97, 95 % CrI 1.26-3.08), the level of education (POR 1.50, 95 % CrI 1.22-1.84), the marital status (POR 1.86, 95 % CrI 1.23-2.80), a higher wealth index (POR 2.11, 95 % CrI 1.77-2.51), the sexual activity (POR 1.76, 95 % CrI 1.04-2.96), and a history of sexually transmitted infection (POR 2.03, 95 % CrI 1.56-2.64).

**Conclusions:**

Our study, which shows where and towards which populations HIV resources should be allocated, could help national health policy makers develop an effective HIV intervention in Burundi. Our findings support the strategy of the Joint United Nations Programme on HIV/AIDS (UNAIDS) for country-specific, in-depth analyses of HIV epidemics to tailor national prevention responses.

## Background

Adequate resource allocation is critical in the battle against HIV/AIDS, especially in sub-Saharan African countries where 70 % of the people living with HIV worldwide currently reside [1]. These countries face major financial constraints, shortages of healthcare workers, and poorly developed healthcare systems [1, 2]. The determination of the location and nature of HIV services to implement must therefore be made according to the state of the epidemic and to the geographic, social and behavioral characteristics of patients. The *‘Know your epidemic’* strategy of the Joint United Nations Programme on HIV/AIDS (UNAIDS) underlines the need for country-specific, in-depth analyses of HIV epidemic features to tailor national prevention responses to the people most at risk [3].

Because half HIV cases only are diagnosed in sub-Saharan Africa [4], most countries rely on Demographic and Health Surveys (DHS) to estimate HIV prevalence along with other data including social and behavioral characteristics [5]. DHS are population-based surveys and use a standardized methodology. They are performed in large, random, clustered samples of people. Since 2001, DHS include informed, anonymous, and voluntary HIV testing in adult women and men. However, a major limitation of crude DHS estimations is that the spatial heterogeneity of HIV prevalence and the hotspots of the disease are hardly examined, although aggregated regional HIV prevalence data can mask large intra-regional differences. In addition, the spatial heterogeneity of HIV prevalence has seldom been taken into account when examining the factors associated with HIV infection.

By contrast to standard statistical tools, spatial analysis methods allow investigating the spatial heterogeneity and identifying the hotspots of diseases independently of administrative boundaries. They also allow accounting for the spatial heterogeneity in the assessment of risk factors. These methods thus provide crucial additional data to national health policy makers for developing effective interventions and allocating financial and human resources based on the local situations. They have therefore been increasingly used in the last years, especially in the field of HIV/AIDS (a disease with a well-known spatial epidemiology) [6–15].

**Fig. 1:**
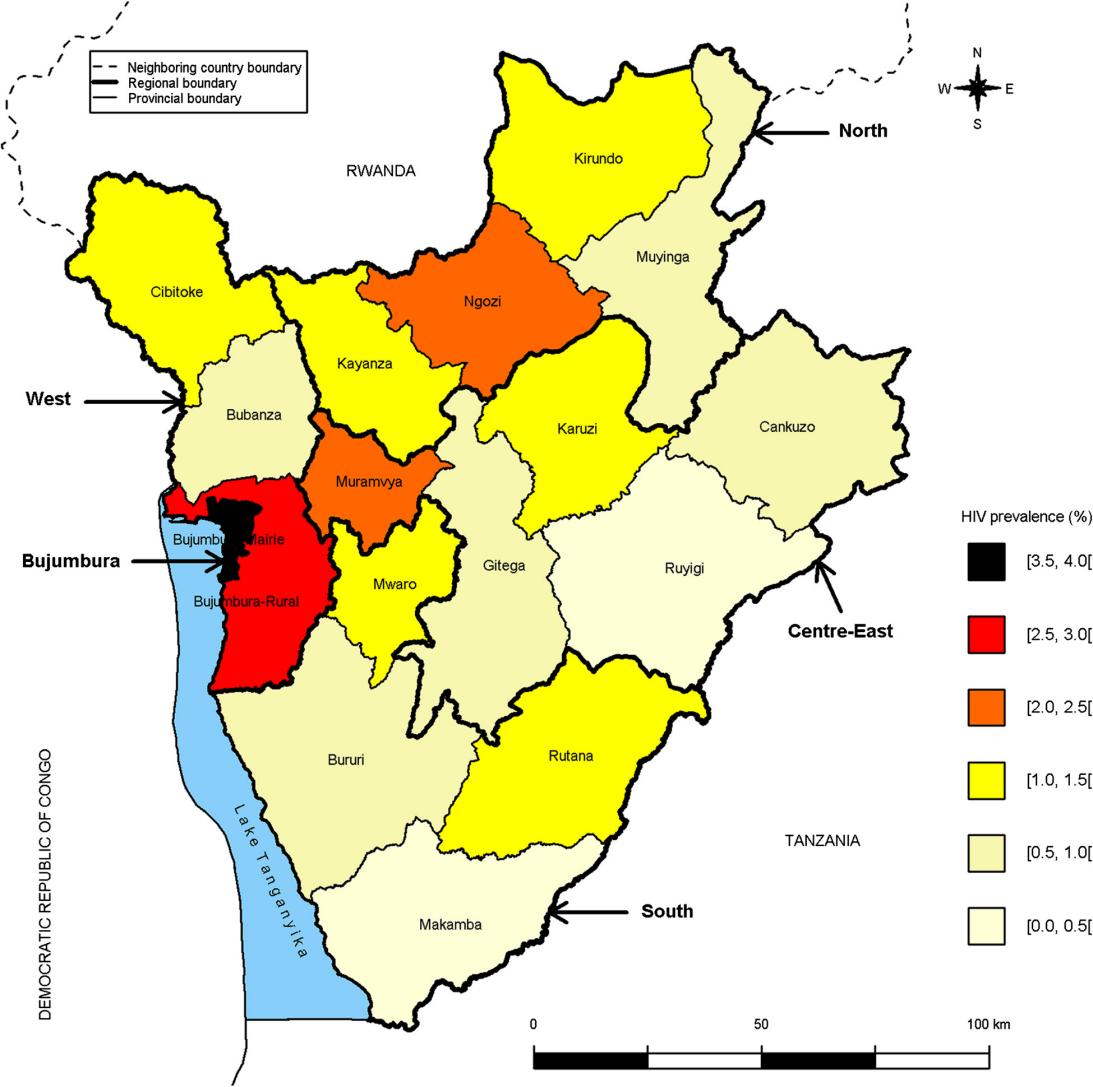
Average HIV prevalence by province in adults aged 15–49 years in Burundi, 2010

Burundi, in Eastern Africa, is among the world’s poorest countries and was severely affected by a civil war from 1993 to 2003. It is bounded by Rwanda to the north, the Democratic Republic of Congo to the west, and Tanzania to the east and south-east (Fig. 1). The population is approximately 11 million inhabitants. The national AIDS program, launched in 1988, includes prevention, testing, care and treatment activities. HIV services have been progressively decentralized to primary health centers throughout the country [16]. The 2010 Burundi DHS reported an overall HIV prevalence of 1.4 % among adults and suggested regional differences with HIV prevalence of 0.9 % in the South, 1.0 % in the Centre-East, 1.3 % in the North, 1.6 % in the West, and 3.7 % in Bujumbura-Mairie (the capital city) [17]. In September 2014, UNAIDS reported estimations of HIV prevalence at the smaller provincial level ranging from 0.4 % in two rural provinces to 3.6 % in Bujumbura-Mairie [18]. Based on individual-level data collected in the 2010 Burundi DHS, we further investigated the spatial heterogeneity of HIV prevalence and then assessed the association of social and behavioral characteristics with HIV infection accounting for the spatial heterogeneity.

## Methods

### Study design

We performed a cross-sectional study based on a secondary analysis of the 2010 Burundi DHS data.

#### Design and procedures of the 2010 Burundi DHS

The 2010 Burundi DHS was conducted by national authorities and ICF International between August 29, 2010 and January 30, 2011 following the standardized DHS methodology [5]. This methodology and crude results are described extensively elsewhere [17]. Briefly, the 2010 Burundi DHS used a stratified two-stage random cluster sampling design. Stratification was made at two levels: the provincial level (17 provinces) and the urban or rural area. At the first stage, 376 of the 8104 enumeration areas (i.e. groupings of households) identified in the 2008 national population and housing census [19] were selected with a probability proportional to their size (i.e. the number of households in each enumeration area). At the second stage, 24 households were selected in each enumeration area with an equal probability, leading to a total of 9024 eligible households. HIV tests were proposed to 50 % of the 9024 households. All women aged 15–49 years and men aged 15–59 years living in or having spent the previous night in one of these households were eligible for HIV testing. The Institutional Review Board of ICF International and the National Ethics Committee of Burundi approved the study protocol. After being provided with information, respondents aged 18 years or older and parents or guardians of minors aged 15–18 years gave their written consent to participate in the survey. Minors gave their oral assent.

Blood spot samples were collected from consenting household residents’ fingers and put on filter papers. Serologic screening for HIV infection was then performed on the dried blood spots at the Public Health National Institute in Bujumbura-Mairie using an enzyme-linked immunosorbent assay (ELISA; Vironostika HIV Uni-Form Ag/Ab, Biomérieux, Marcy l’Etoile, France). All positive samples and 10 % of the negative samples (for quality control) were further tested with a second ELISA (Enzignost HIV Integral II, Siemens, Erlangen, Germany). Samples with discordant results between both tests were reanalyzed using a line immunoassay (INNO-LIA HIV I/II Score, Innogenetics, Gent, Belgium).

Social and behavioral characteristics of residents were collected using a standardized questionnaire and included gender (woman or man), age (continuous variable), level of education (no formal education, primary, secondary, or superior), marital status (single, married, cohabiting, divorced, separated, or widowed), religion (Catholic, Protestant, Muslim, Adventist, Jehovah’s witness, other, or none), wealth quintile (poorest, poorer, middle, richer, or richest), sexual activity (never had sex, active in the last four weeks, or not active in the last four weeks), the number of extramarital sex partners in the last 12 months, and a history of sexually transmitted infection (STI) in the last 12 months (yes or no).

The geographic coordinates (latitude and longitude) of the 376 enumeration areas were collected using the geographic information system (GIS) and global positioning system (GPS) technologies. They were recorded at the center of the enumeration areas. In order to preserve the confidentiality of the respondents, the GPS latitude/longitude positions were randomly displaced (up to 2 km for the urban enumeration areas and up to 5 km for the rural enumeration areas, with 1 % of the rural enumeration areas being displaced up to 10 km).

### Statistical analysis

#### Study population

For the present study, the analyses were restricted to the 15–49 year age group because HIV testing had not been performed in women aged 50–59 years (by contrast to men) in the 2010 Burundi DHS. It is worth noting that UNAIDS also reports adult data in the 15–49 year age group [1, 18].

#### Data

All analyses used the individual-level data and their specific weights provided in the DHS databases (available from the DHS program website [5]). As usual, the weights took into account the survey design of the 2010 Burundi DHS and the proportion of respondents in each enumeration area. They were equal to the inverse of the probability for a given resident of being included in the survey.

#### Spatial heterogeneity of HIV prevalence

First, we computed and mapped the crude estimations of HIV prevalence in the 17 provinces. We then used a non-spatial logistic regression model to assess the relationship between HIV infection and the provinces, taking the province with the lowest HIV prevalence as the reference category. These analyses were performed using the Stata software version 11 [20].

Second, we analyzed the spatial autocorrelation of HIV prevalence data by performing a global Moran test. Moran’s I statistic tests the null hypothesis that observed data at one location are independent of data at other locations. Its value ranges from −1 (data perfectly dispersed), 0 (data randomly dispersed) to 1 (data perfectly correlated). Because the Moran’s I statistic showed the existence of a significant spatial autocorrelation of our HIV prevalence data, we subsequently analyzed the data using a geostatistical approach which takes into account this spatial autocorrelation.

Third, we mapped HIV prevalence throughout the country independently of provincial boundaries using a Gaussian kernel density estimation with adaptive bandwidths and the specific prevR package of R software (R Core Development Team, April 10, 2014) [21, 22]. This approach is promoted by UNAIDS and has been used to estimate HIV prevalence at a sub-national level in various countries including Burundi [18, 23]. It allows generating a smoothed surface of HIV prevalence based on observed data. In our study, we interpolated HIV prevalence data in 449,065 points using the observed data in the 376 enumeration areas. As recommended by Larmarange and Bendaud [23], we set the number of observations at 500 so that the bandwidths adapt to capture this minimum number.

Finally, we identified the spatial clusters with high and low HIV prevalence using the Kulldorff spatial scan statistics (SaTScan software version 9.3) [24]. This method has been widely used in the last years, especially in the field of HIV/AIDS [7, 10, 12, 15, 25–27]. It allows finding the location of areas with higher or lower numbers of HIV cases than expected under the hypothesis of uniform spatial distribution of cases by gradually scanning circular windows of various sizes across the study area. We assumed that the number of HIV cases in each circular window was an independent Bernoulli random variable. For the circular windows, we used a maximum radius of 15 Km for the detection of clusters with high HIV prevalence and of 50 Km for the detection of clusters with low prevalence. We chose these radii because high HIV prevalence was more likely in small, densely populated areas such as in Bujumbura-Mairie where the maximum distance between the centroid and the city’s boundaries is of 13 Km while low HIV prevalence was more likely in large, sparsely populated areas. We also used the default value of 50 % of the total study population for the maximal size of the clusters. The statistical significance of clusters was ascertained using the likelihood ratio test and its associated *p*-value obtained through 999 Monte Carlo simulations. The null hypothesis of uniform spatial distribution of HIV cases (no cluster) was rejected if the *p*-value was <0.05. When a cluster was identified, the strength of the clustering was estimated using the relative risk of excess HIV cases.

#### Factors associated with HIV infection

The association of social and behavioral characteristics with HIV infection (infected or not) was investigated using a spatial logistic regression model performed with the BayesX software version 2.1 [28]. This model allowed adjusting for the spatial and non-spatial random effects of provinces. The parameters were estimated using 400 Markov chain Monte Carlo simulations in restricted maximum likelihood regression models. Independent covariates associated with HIV infection with a conservative *p*-value of <0.2 in univariate analysis were subsequently tested in multivariate analysis [29]. A backward elimination procedure was used to determine the final model containing only the covariates significantly associated with HIV infection. The strength of associations was estimated using the posterior odds ratios (PORs) and their 95 % credible intervals (CrIs). Finally, the goodness-of-fit of models was assessed using the conditional Bayesian Information Criterion (BIC).

All statistical tests were interpreted at the 0.05 significance level.

## Results

### Characteristics of the study population

Of 9503 residents eligible for HIV testing, 90.4 % were interviewed and tested (91.8 % of 4911 eligible women and 88.8 % of 4592 eligible men). Five hundred and one men, aged 50–59 years, were excluded from the present analysis. After weighting, 8086 residents aged 15–49 years enrolled in 3816 households from the 376 enumeration areas were included in the analysis. Median number of residents by enumeration area was 21 (interquartile range [IQR] 18–25). There were 4532 women and 3554 men (Table 1). Median age was 26 years (IQR 20–35). Most residents had attended at least primary school (62.1 %), were not single (62.4 %), were Catholic (62.7 %), had sex in the last 4 weeks (51.7 %), had no extramarital sex partner in the last 12 months (96.7 %), and had no STI in the last 12 months (98.1 %). There were 114 HIV cases (78 women and 36 men), leading to an overall HIV prevalence of 1.4 %.

**Table 1:** HIV prevalence by province and resident’s characteristics in adults aged 15–49 years in Burundi, 2010

	Number^*^	HIV + ^*^	Percent
Province			
Bubanza	379 (4.7 %)	3	0.9
Bujumbura-Mairie	731 (9.0 %)	27	3.7
Bujumbura-Rural	493 (6.1 %)	13	2.7
Bururi	654 (8.1 %)	6	0.9
Cankuzo	209 (2.6 %)	2	1.0
Cibitoke	489 (6.0 %)	5	1.1
Gitega	590 (7.3 %)	3	0.6
Karuzi	439 (5.4 %)	5	1.1
Kayanza	560 (6.9 %)	6	1.1
Kirundo	547 (6.8 %)	8	1.4
Makamba	474 (5.9 %)	2	0.3
Muramvya	313 (3.9 %)	8	2.4
Muyinga	610 (7.5 %)	5	0.8
Mwaro	288 (3.6 %)	4	1.3
Ngozi	613 (7.6 %)	13	2.2
Rutana	334 (4.1 %)	4	1.3
Ruyigi	362 (4.5 %)	1	0.1
Gender			
Women	4532 (56.0 %)	78	1.7
Men	3554 (44.0 %)	36	1.0
Age group (years)			
15–19	2014 (24.9 %)	5	0.3
20–24	1544 (19.1 %)	14	0.9
25–29	1341 (16.6 %)	13	0.9
30–34	962 (11.9 %)	20	2.1
35–39	864 (10.7 %)	24	2.8
40–44	702 (8.7 %)	23	3.3
45–49	659 (8.1 %)	15	2.3
Level of education			
No formal education	3067 (37.9 %)	34	1.1
Primary school	3706 (45.8 %)	61	1.6
Secondary school	1197 (14.8 %)	17	1.4
Superior	116 (1.5 %)	2	1.7
Marital status			
Single	3040 (37.6 %)	11	0.4
Married	3272 (40.5 %)	39	1.2
Cohabiting	1329 (16.4 %)	40	3.0
Widowed	201 (2.5 %)	16	8.1
Divorced or separated	243 (3.0 %)	6	2.6
Religion			
Catholic	5066 (62.7 %)	59	1.2
Protestant	2359 (29.2 %)	36	1.5
Muslim	204 (2.5 %)	10	4.7
Adventist	185 (2.3 %)	3	1.5
Jehovah’s Witnesses	20 (0.3 %)	1	5.3
Other	63 (0.8 %)	1	2.2
None	180 (2.2 %)	4	2.1
Wealth index			
Poorest	1469 (18.2 %)	17	1.2
Poorer	1558 (19.3 %)	16	1.0
Middle	1583 (19.6 %)	20	1.2
Richer	1645 (20.3 %)	12	0.7
Richest	1831 (22.6 %)	49	2.7
Sexual activity			
Never had sex	2457 (30.4 %)	5	0.2
Active in the last 4 weeks	4177 (51.7 %)	68	1.6
Not active in the last 4 weeks	1446 (17.9 %)	41	2.8
Number of extramarital sex partners in the last 12 months			
0	7815 (96.7 %)	106	1.4
1	238 (2.9 %)	7	2.8
≥ 2	29 (0.4 %)	1	4.6
Sexually transmitted infections in the last 12 months			
No	7915 (98.1 %)	102	1.3
Yes	153 (1.9 %)	12	7.8
Total	8086 (100.0 %)	114	1.4

^*^Weighted numbers were rounded

### Spatial heterogeneity of HIV prevalence

The crude estimations of HIV prevalence ranged from 0.1 % in the Ruyigi province to 3.7 % in Bujumbura-Mairie (Fig. 1 & Table 1). HIV prevalence was also high in the Bujumbura-Rural (2.7 %), Muramvya (2.4 %) and Ngozi (2.2 %) provinces. The logistic regression analysis further showed that HIV infection was significantly more frequent in six provinces (Bujumbura-Mairie, Bujumbura-Rural, Kirundo, Muramvya, Ngozi and Rutana) than in the Ruyigi province (Table 2).

**Table 2:** Relationship between HIV infection and provinces in adults aged 15–49 years in Burundi, 2010

Province	OR	95 % CI	p*
Bubanza	6.32	0.60–66.50	0.124
Bujumbura-Mairie	28.07	3.63–217.04	0.001
Bujumbura-Rural	20.41	2.42–172.16	0.006
Bururi	6.22	0.63–61.17	0.117
Cankuzo	6.97	0.74–65.74	0.090
Cibitoke	7.85	0.79–77.69	0.078
Gitega	4.18	0.34–51.37	0.263
Karuzi	8.03	0.89–72.14	0.063
Kayanza	7.90	0.89–70.33	0.064
Kirundo	10.16	1.13–91.22	0.039
Makamba	2.44	0.23–25.38	0.456
Muramvya	18.12	2.21–148.54	0.007
Muyinga	5.52	0.48–63.60	0.170
Mwaro	9.23	0.89–95.74	0.063
Ngozi	16.17	1.86–140.79	0.012
Rutana	9.72	1.08–87.44	0.043
Ruyigi	1.00		

*OR* odds ratio, *CI* confidence interval. *P-value for the corresponding province versus the Ruyigi province

**Fig. 2:**
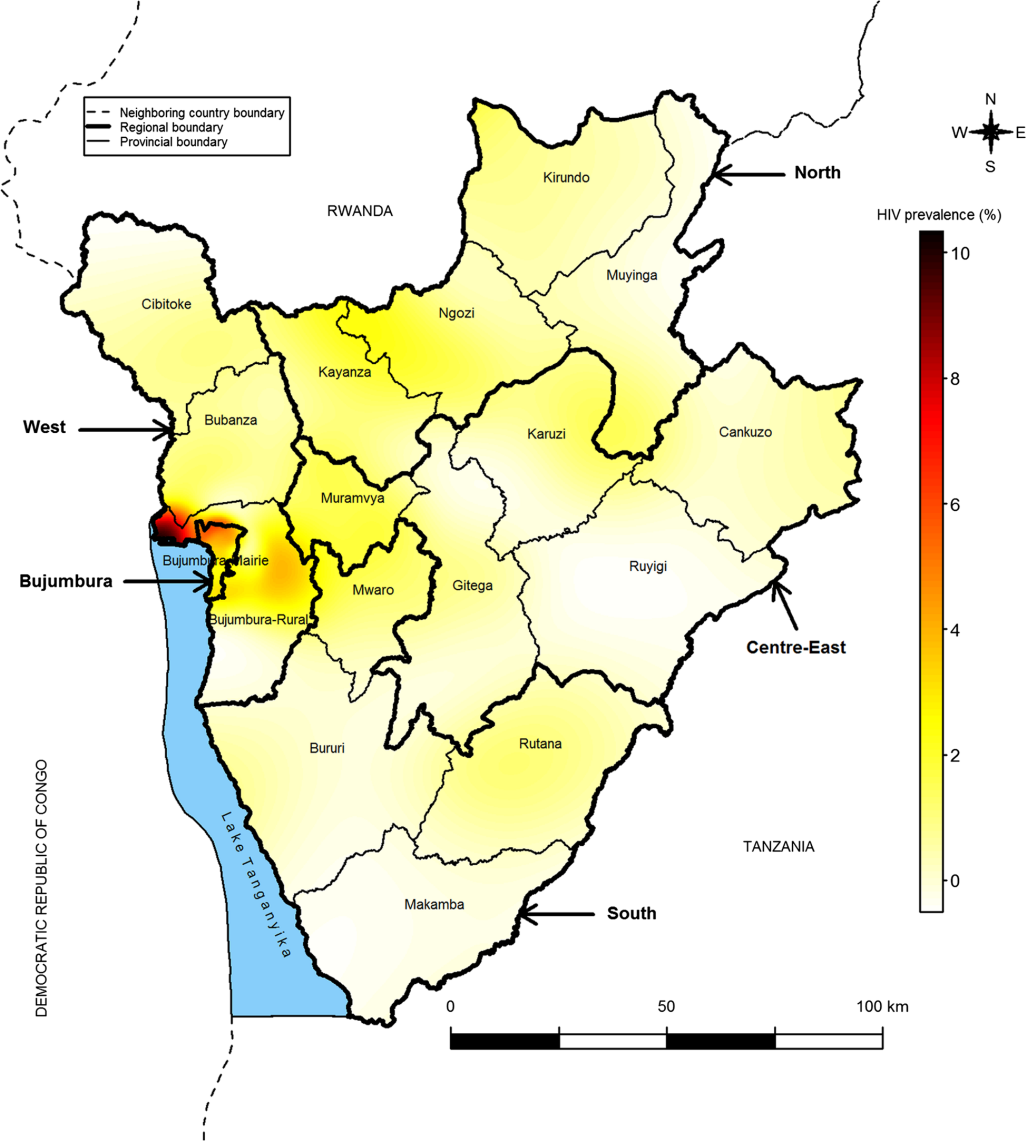
Interpolated HIV prevalence in adults aged 15–49 years in Burundi, 2010

Data of HIV prevalence were spatially auto correlated (Moran’s I = 0.03, *p* = 0.021). The interpolated data showed the great spatial heterogeneity of HIV prevalence (from 0 to 10 %), independently of provincial boundaries (Fig. 2). The higher HIV prevalence was observed near Bujumbura-Mairie. Prevalence above 2.0 % was also observed in different locations throughout the country. By contrast, HIV prevalence was especially low in the Centre-East and South regions.

**Fig. 3:**
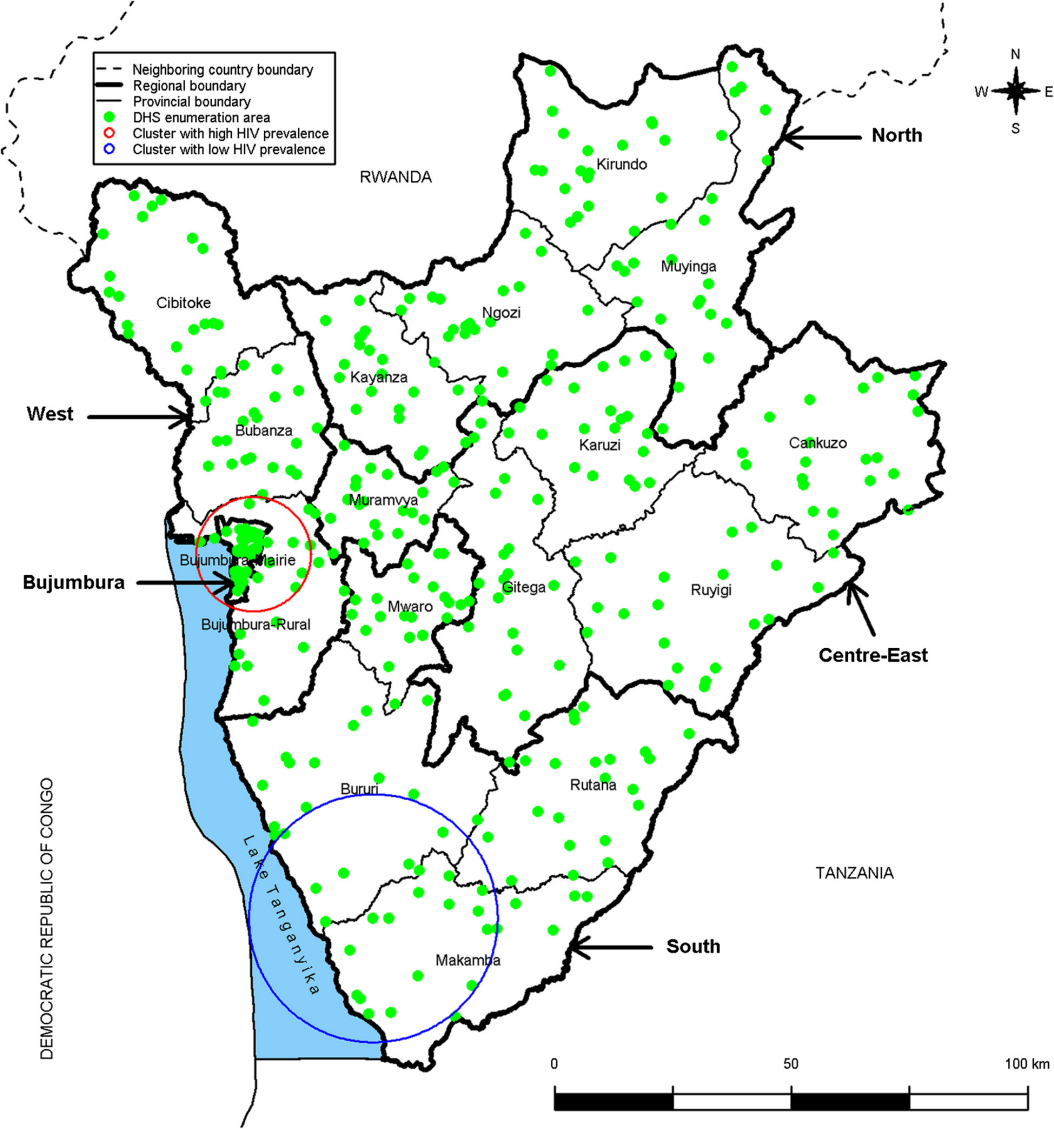
Spatial distribution of the clusters with high and low HIV prevalence in adults aged 15–49 years in Burundi, 2010

The spatial scan statistics analysis confirmed these findings (Fig. 3). It identified a cluster with high HIV prevalence with a 13.5 Km radius in Bujumbura-Mairie and adjacent areas. There were 37 HIV cases (32.5 % of all) giving a relative risk of 3.7 (*p* < 0.001). HIV prevalence was thus 3.9 % among the 943 residents from 50 enumeration areas. The spatial scan statistics analysis also identified a cluster with low HIV prevalence with a 29.1 Km radius which straddled the Makamba and Bururi provinces (*p* = 0.02). No HIV case was detected in this cluster although the study included 651 residents from 23 enumeration areas. By contrast, no cluster was identified in the Centre-East region including the Ruyigi province.

### Spatial analysis of factors associated with HIV infection

After controlling for the spatial heterogeneity (Table 3), HIV infection was strongly associated in the univariate analysis with the female sex (POR 1.43, 95 % CrI 1.20-1.70, *p* < 0.001), an older age (e.g. POR 2.96, 95 % CrI 2.04–4.28, *p* < 0.001 for 35–39 years versus 15–19 years), the marital status (e.g. POR 4.31, 95 % CrI 3.18–5.86, *p* < 0.001 for widowed versus single), a higher wealth index (POR 1.98, 95 % CrI 1.67-2.35, *p* < 0.001 for richest versus others), the sexual activity (e.g. POR 2.68, 95 % CrI 1.86–3.85, *p* < 0.001 for active in the last 4 weeks versus never had sex), and a history of STI in the last 12 months (POR 2.55, 95 % CrI 1.98–3.30, *p* < 0.001). HIV infection also tended to be associated with the religion (POR 1.85, 95 % CrI 0.99–3.44, *p* = 0.052 for Muslim versus Catholic), and the number of extramarital sex partners in the last 12 months (POR 1.76, 95 % CrI 0.98-3.15, *p* = 0.057 for ≥2 extramarital sex partners versus none) but the statistical significance was not reached. By contrast, HIV infection was not associated with the level of education (POR 1.15, 95 % CrI 0.95–1.38, *p* = 0.141).

**Table 3:** Univariate and multivariate spatial logistic regression analyses of factors associated with HIV infection in adults aged 15–49 years in Burundi, 2010

	Univariate			Multivariate		
	POR	95 % CrI	*p*	aPOR	95 % CrI	*p*
Gender						
Men	1.00			1.00		
Women	1.43	1.20–1.70	<0.001	1.36	1.13–1.64	0.001
Age group (years)						
15–19	1.00			1.00		
20–24	1.59	1.06–2.37	0.024	1.19	0.77–1.85	0.425
25–29	1.85	1.25–2.74	0.003	1.21	0.76–1.91	0.420
30–34	2.44	1.67–3.57	<0.001	1.56	0.99–2.45	0.056
35–39	2.96	2.04–4.28	<0.001	1.97	1.26–3.08	0.004
40–44	2.99	2.04–4.38	<0.001	2.14	1.34–3.42	0.002
45–49	3.06	2.09–4.50	<0.001	2.13	1.32–3.44	0.002
Level of education						
No formal education	1.00			1.00		
Primary school or higher	1.15	0.95–1.38	0.141	1.50	1.22–1.84	<0.001
Marital status						
Single	1.00			1.00		
Married	1.71	1.32–2.21	<0.001	0.91	0.61–1.34	0.622
Cohabiting	2.56	1.97–3.32	<0.001	1.46	0.99–2.15	0.057
Widowed	4.31	3.18–5.86	<0.001	1.86	1.23–2.80	0.004
Divorced or separated	3.08	2.17–4.36	<0.001	1.56	1.04–2.36	0.033
Religion						
Catholic	1.00					
Protestant	1.00	0.55–1.80	0.999			
Muslim	1.85	0.99–3.44	0.052			
Adventist	1.18	0.55–2.53	0.661			
Jehovah’s Witnesses	1.94	0.79–4.80	0.150			
Other	1.30	0.53–3.18	0.570			
None	0.91	0.51–1.63	0.757			
Wealth index						
Richest	1.98	1.67–2.35	<0.001	2.11	1.77–2.51	<0.001
Others	1.00			1.00		
Sexual activity						
Never had sex	1.00			1.00		
Active in the 4 last weeks	2.68	1.86–3.85	<0.001	1.76	1.04–2.96	0.034
Not active in the 4 last weeks	3.50	2.42–5.06	<0.001	2.10	1.32–3.35	0.002
Number of extramarital sex partners in the last 12 months						
0	1.00					
1	1.13	0.78–1.62	0.515			
≥ 2	1.76	0.98–3.15	0.057			
Sexually transmitted infections in the last 12 months						
No	1.00			1.00		
Yes	2.55	1.98–3.30	<0.001	2.03	1.56–2.64	<0.001

*POR* posterior odds ratio, *CrI* credible interval

By multivariate spatial analysis (Table 3), HIV infection remained significantly associated with the female sex (POR 1.36, 95 % CrI 1.13-1.64, *p* = 0.001), an older age (e.g. POR 1.97, 95 % CrI 1.26–3.08, *p* = 0.004 for 35–39 years versus 15–19 years), the marital status (e.g. POR 1.86, 95 % CrI 1.23-2.80, *p* = 0.004 for widowed versus single), a higher wealth index (POR 2.11, 95 % CrI 1.77-2.51, *p* < 0.001 for richest versus others), the sexual activity (e.g. POR 1.76, 95 % CrI 1.04-2.96, *p* = 0.034 for active in the last four weeks versus never had sex), and a history of STI in the last 12 months (POR 2.03, 95 % CrI 1.56-2.64, *p* < 0.001). HIV infection was also significantly associated with the level of education, being higher in residents who had attended school than in those who did not (POR 1.50, 95 % CrI 1.22–1.84, *p* < 0.001).

## Discussion

This spatial study allowed identifying populations at higher risk of HIV infection because of geographic, social or behavioral characteristics in Burundi.

Thus, our study added important information on the spatial heterogeneity of HIV infection in this country. We first found a significant association between HIV infection and the provinces. By contrast, the 2010 Burundi DHS report did not analyze HIV data by province [17]. UNAIDS recently reported HIV prevalence by province but did not assess the statistical significance of differences [18]. Our interpolated data further showed different locations with relatively high HIV prevalence, independently of provincial boundaries. Finally, we identified a cluster with high HIV prevalence centered in Bujumbura-Mairie and a cluster with low prevalence in the southern part of the country. Cuadros et al. also found a significant cluster with high HIV prevalence in Bujumbura-Mairie but they did not detect any significant cluster with low prevalence (*p* = 0.069) probably because they performed their study in a slightly different population including men aged 50–59 years and used unweighted data [7]. Overall, our findings in Burundi are consistent with the localized spatial clustering of HIV infection found in other countries [6–10, 15]. They highlighted that the crude regional estimations of the 2010 Burundi DHS report masked intra-regional heterogeneities in HIV prevalence [17].

In our study controlling for this spatial heterogeneity, HIV infection was significantly more frequent in the residents who were women, older than 35 years, educated, widowed, divorced or separated, richest, sexually active, and in those who had had STIs in the last 12 months. These factors are well-known risk factors for HIV infection [30–34]. However, the novelty of our spatial analysis is that it provided better estimators of the strength of associations than a non-spatial analysis because the former takes into account the spatial autocorrelation of data.

Our findings confirmed that in-depth analyses of local HIV epidemics are crucial for national AIDS programs when designing the most effective prevention responses [3]. Indeed, the reduction of the number of new HIV infections implies the need for a greater understanding of *‘where’* and *‘towards which populations’* efforts should be concentrated, in terms of primary and secondary prevention activities such as counseling, availability and accessibility of condoms, HIV testing, linkage to care, early antiretroviral treatment, and support. For instance in Burundi, our findings suggest that HIV activities should be especially reinforced in and around Bujumbura-Mairie. With regard to social or behavioral characteristics, greater efforts should be focused on higher risk groups such as women, people who are older than 35 years, educated, widowed, divorced or separated, richest, sexually active, and those with STIs. In addition, the reasons for the spatial heterogeneity of HIV prevalence should be investigated.

One of the strengths of our study was the use of data collected in a DHS based on a standardized methodology. A second strength was that this DHS was large, involving 8086 residents enrolled in 3816 households from 376 enumeration areas. Thus, although the 2010 Burundi DHS was designed to provide estimates of HIV prevalence at the national and regional level (as the other DHS), estimates at the provincial level have been found to be good or moderately good [18, 23]. Finally, we analyzed data using standardized geostatistical methods which take into account the spatial autocorrelation of data.

Our findings should be interpreted taking into account several study limitations. First, as HIV prevalence is quite low in Burundi, the spatial variability was relatively modest. In addition, there was zero HIV case in certain enumeration areas. This may have limited our ability to find significant associations with HIV infection at the provincial level (for instance, between the Cibitoke, Karuzi, Kayanza and Mwaro provinces and the Ruyigi province). Second, the spatial logistic regression method used here to assess the association of social and behavioral characteristics with HIV infection provides one single posterior odds ratio by characteristic assuming that the strength of the association is uniform over the study area. However, this hypothesis might be incorrect, especially as our study area was large (the whole country). An additional analysis using a geographically weighted regression method which would provide the posterior odds ratios specific to the cluster with high HIV prevalence might be useful for identifying the populations most in need of interventions [35].

## Conclusion

In conclusion, this study could help health policy makers develop an effective intervention in Burundi by showing where and towards which populations HIV resources should be allocated. Our findings support the need for in-depth analyses of HIV epidemics in every countries to tailor national prevention responses, as promoted by UNAIDS. This should encourage program managers in other countries to perform such studies in their own settings. This kind of study is not costly and is relatively rapid thanks to the availability of recurrent DHS data.

## Abbreviations

AIDS: acquired immune deficiency syndrome
CrI: credible interval
DHS: demographic and health survey
GIS: geographic information system
GPS: global positioning system
HIV: human immunodeficiency virus
IQR: interquartile range
POR: posterior odds ratio
STI: sexually transmitted infection
UNAIDS: Joint United Nations Programme on HIV/AIDS
